# More questions than answers! Clinical dilemmas in psychopharmacology in pregnancy and lactation

**DOI:** 10.4103/0019-5545.44901

**Published:** 2009

**Authors:** Geetha Desai, Girish N. Babu, Ravi P. Rajkumar, Prabha S. Chandra

**Affiliations:** Department of Psychiatry, NIMHANS, Bangalore - 560029, India

**Keywords:** Psychotropics, pregnancy, lactation

## Abstract

Women in childbearing age frequently suffer from mental illness. Maternal psychiatric disorders may have a devastating impact on the fetus and the newborn. Thus treating or preventing relapse of these disorders during pregnancy and puerperium is a clinical and ethical duty with the necessity to avoid or minimize fetal or neonatal drug exposure. Though there are many guidelines and comprehensive reviews regarding drug safety in pregnancy and lactation, the application of these recommendations into clinical practice appears to be complex. Hence, we present some clinical questions with answers considering the available literature on safety of psychotropics in pregnancy and lactation.

## INTRODUCTION

Psychiatric disorders during pregnancy and the postpartum period are common. Many women are likely to require treatment for the same during pregnancy and while breast-feeding. Clinicians are often apprehensive about treating pregnant and lactating women, as the information on safety of psychotropics is either inconclusive or undetermined. With the advent of newer psychotropics, often minimal data is available and one has to continuously keep pace with newer information on drug safety. Multiple issues have to be considered while choosing safe treatments for pregnant and lactating women and the best approach is to individualize treatment. We have discussed several challenges commonly faced by the clinician in this situation. The clinical dilemmas discussed in this paper have been compiled based on real life clinical scenarios faced at the Perinatal Psychiatry services, NIMHANS, Bangalore and questions commonly asked by trainees and practicing psychiatrists. The answers have been formulated based on the latest available evidence in published literature.

**A lady who has been diagnosed to have bipolar disorder has been taking prophylactic lithium for the past 2 years. She has had multiple relapses in the past due to drug discontinuation. She is now 3 months pregnant and has stopped Lithium since 10 days following positive pregnancy test. What do I do in this situation?**

Lithium is a commonly used mood stabilizer in bipolar disorder and has evidence of teratogenicity. The risk estimate for cardiovascular malformations including Ebstein's anomaly, during first trimester exposure to lithium, is between 0.05% and 0.15 or 10-20 times the risk in the general population.[1] However the absolute risk with lithium exposure remains low (1 in 1000 births).[2,3] Since there has been first trimester exposure to lithium, this lady should be advised the option of high resolution ultrasound and a fetal echocardiogram at 18–20 weeks gestation to detect cardiac abnormalities. Although early reports suggested that pregnancy was protective against mood episodes, recent data indicate that bipolar women who are pregnant experience mood episodes at rates similar to those who are not pregnant.[4,5] Hence, a discussion regarding the risks and benefits of discontinuation of lithium should be done. There are reports of neonatal toxicity in neonates born to women on lithium during labor and delivery.[6] Data on long-term neurobehavioral sequelae is limited, though available data does not indicate any long term effects.[7] Discuss the maximum risk period for cardiac anomaly and role of lithium /antipsychotic as a mood stabilizer as she has had multiple relapses in the past.

**A 28-year-old lady has presented in her 10th week of pregnancy and has been taking Valproate 1000 mg since 8 months. An ultrasound scan shows increased nuchal translucency. She is in a dilemma: should she continue the pregnancy or not? How do I proceed?**

Sodium Valproate has been considered a human teratogen. Use of sodium Valproate during the first trimester is associated with neural tube defect at the rate of 1%–5% and the risk is dose related.[8,9] Even exposure late in pregnancy is associated with craniofacial abnormalities and cognitive dysfunction.[10–12] This woman should be advised to undergo an ultrasound at 16–19 weeks of gestation to detect neural tube defects, followed if necessary, by amniocentesis for detection of spina bifida. Once this is confirmed, a final decision regarding continuation or termination of pregnancy should be taken by the mother after being given appropriate information. Discuss folate supplementation.

**I have been treating a lady with bipolar disorder stabilized on lithium and olanzapine. She has taken lithium throughout her pregnancy and has delivered a male child. She wants to breast feed the baby. What should I advice?**

Lithium is secreted in breast milk. The American Academy of Pediatrics has categorized lithium as a drug that “should be given to nursing mothers with caution.”[13] The concentration of lithium in the serum of nursing infants is approximately one-tenth to one-half of the mother's serum concentrations.[14–16] There is concern regarding rapid dehydration in neonates if they develop a febrile illness that could cause lithium levels in the infant to increase. The long-term effects on infants with sustained lithium levels are unknown. It is important to monitor lithium serum concentration and administer a complete blood panel including thyroid hormone levels, blood urea, serum creatinine in the breast fed infant. In the Indian setting, such monitoring may be difficult and hence lithium may not be compatible with breast-feeding. The safety of olanzapine in lactation is discussed elsewhere.

**A 25-year-old woman has been diagnosed to have post partum mania. She was started on olanzapine but has shown poor response. She had earlier responded well to Valproate. What should I advice regarding breast-feeding if Valproate is being initiated?**

Valproate has been classified as compatible with breast feeding by the American Academy of Pediatrics, supported by the American Academy of Neurology.[13,17] No adverse effects to infants have been reported when mothers are treated solely during breast feeding.[6,18,19] One case report of thrombocytopenic purpura and anemia has been reported in a infant whose mother used Valproate during pregnancy and lactation, which resolved after discontinuation of breast feeding.[20] Some experts have raised concerns of hepatotoxicity in infants with the use of Valproate.[18] It may be recommended to monitor the clinical status of infant, liver enzymes and platelets in the breast fed infant.[21,22]

**A 22-year-old lady who has early onset bipolar disorder and has been stabilized on carbamazepine, lithium and risperidone since one year. In the past, she has had multiple severe manic episodes on drug default. She is married and wants to conceive. She wants to know if she can stop medication. What do I advise?**

Use of carbamazepine in the first trimester is associated with teratogenicity. The rate for neural tube defects ranges between 0.5% and 1%.[23,24] Other anomalies include craniofacial defects and fingernail hypoplasia.[25] Polytherapy may increase the risk of teratogenicity. It is advisable to maintain the least teratogenic drug with the lowest effective dose and folate supplementation (5 mg per day). Other issues related to lithium and risperidone discussed elsewhere. It is important to decide which drug is least teratogenic for the infant without compromising the mother's mental state. In this case you might consider tapering Carbamazepine and continuing lithium and risperidone after discussion with the woman regarding advantage and disadvantages of this regime. Folate supplementation is important as some women may get pregnant while tapering is going on.

**I have been treating a 30-year-old patient with schizophrenia who is on Depot Flupenthixol 40 mg fortnightly. She has just found out that she is 6 weeks pregnant. What is the correct advice to give her?**

Safety of flupenthixol in pregnancy has not been established though no major malformations have been reported. However, it has been recommended that depot neuroleptics should be avoided in pregnancy unless absolutely essential.[26] If your patient is compliant with medication, you should consider an antipsychotic whose safety profile is well established such as haloperidol. Alternatively, after explaining the risks and uncertainties, you might want to continue oral flupenthixol. You must, however, get an anomaly scan (ultrasound) done at 16 weeks to ensure that there are no major problems. The lowest possible dose of antipsychotic is preferred and you must avoid polytherapy (i.e., using more than one type of drug).

**My patient is taking 300 mg of clozapine and has responded to it after a failed trial of risperidone and quetiapine. She wants to have a baby now. What is the correct treatment protocol?**

The safety of clozapine in pregnancy has not been established. Novartis at Basle, Switzerland, through its pharmacovigilance and epidemiology service has data on nearly 200 cases and reported a 6% rate of malformations.[27] This data must be considered with caution since they represent only the pregnancies reported spontaneously to the pharmaceutical company. No major complications during labor have been reported. Case reports have not reported any developmental delay among infants and toddlers exposed to clozapine in utero. In a review, Dev and Krupp[28] reported on 61 children born to 59 women who received clozapine, 51 of the children were healthy, 5 had congenital anomalies and 5 had perinatal syndromes.

Plasma concentrations of clozapine may diminish during pregnancy due to a higher hepatic metabolism and distribution volume and may lead to exacerbation of psychosis. Being overweight, having glucose intolerance or a family history of diabetes are risk factors to develop gestational diabetes with clozapine. The clinical protocol of any woman taking an atypical antipsychotic during pregnancy should include monitoring of the blood glucose and body weight. Nguyen and Lalonde (2003)[27] conclude in their review that no specific risks for the mother and children can be attributed to the use of clozapine during pregnancy. However, the plasma concentration of clozapine is higher in the fetus compared to the mother; therefore, a Minimal effective dosage should be used. Since clozapine is present in maternal milk, breast feeding should be avoided. The advantages to use clozapine during pregnancy must exceed the risks.

If the woman decides to continue clozapine, use the Lowest effective dose, check for impaired glucose tolerance, record body weight and add 5 mg of folic acid per day to protect from neural tube defects due to obesity. If on the other hand, she decides to stop clozapine, do a gradual cross over with a Safer antipsychotic like haloperidol or risperidone, monitor for worsening of symptoms and keep the patient on close follow up.

**I have been treating a 34-year-old woman with schizophrenia who is on 15 mg of Olanzapine and is two months pregnant. She is mildly overweight but is clinically stable for the last one year on this dose. Is it alright for her to continue olanzapine? What precautions can we follow to minimize adverse effects?**

Olanzapine has not been reported to have any major teratogenic effects till date; however, the risks seem to be related to weight gain and gestational diabetes. Reports of large for date babies have also been published.[29]

If your patient is clinically stable, then check for history of diabetes in an earlier pregnancy and/or a family history of diabetes. Get regular blood glucose levels done, especially after the 20th week. Even impaired glucose tolerance may be associated with large for date babies; hence, regular monitoring and discussion with an obstetrician and physician is needed. As mentioned above (with clozapine), folic acid supplementation is required. Try to maintain the lowest possible dose of the drug. Olanzapine has been reported to be safe in breast feeding.

**My patient with bipolar disorder has just had a baby. She is taking risperidone 4 mg as mood stabilizers were stopped during pregnancy. She is breast feeding her baby. What advice can I give her?**

There is limited data about the safety of risperidone during breast feeding. The recommendation for safe breast-feeding is that the ratio of infant dose exposure to maternal dose not be greater than 10%. Hill *et al.*[30] calculated the estimated infant dose exposure during breast-feeding and found that both olanzapine and risperidone were well below the “attention critical” concentration.

Hence, it appears that you may continue risperidone at the lowest possible dose and monitor the infant for side effects such as drowsiness or extra pyramidal symptoms. If the infant is premature, one has to be more cautious because of hepatic immaturity.

**A 20-year-old female was brought to the emergency ward with a history of violent behavior since 2 weeks. She was diagnosed to have Acute psychosis and also found to be 5 months pregnant. The resident was concerned about the initial control of agitation; hence, the resident wanted to take an opinion from his senior colleagues before using parenteral drugs to control her agitation.**

There are no studies that address the pharmacological management of agitation during pregnancy. The only attempt to address this issue is the Clinical Consensus Guidelines published in 2001,[31] which recommends the use of haloperidol for agitation in Pregnant women. A high-potency conventional antipsychotic was rated as the first-line response by 76% of the consensus panel. Although no consensus was reached on second-line treatment, the top three recommendations were as follows: 40%, benzodiazepine alone; 33%, risperidone alone; and 29%, a combination of a benzodiazepine and a high-potency conventional antipsychotic. In a retrospective study by Ladavac *et al.*[32] on 80 pregnant women, lorazepam was the only benzodiazepine, while various antipsychotics including haloperidol, risperidone, ziprasidone and quetiapine were used for acute control of agitation. Intramuscular haloperidol was the most frequently administered followed by oral risperidone. Physical restraints may pose significant risks to the pregnant patient particularly in the second- and third-trimesters, due to obstruction of venous return to the heart and supine hypertension syndrome if placed in the supine position. The fetal risk of using several doses of psychotropic medication to treat agitated pregnant women in the emergency setting remains unknown. Hence in the above clinical situation haloperidol IM or oral risperidone can be used to treat agitation.

**A 25-year-old female presents with a history of recurrent depressive disorder and has been taking fluoxetine 20 mg/day. She has come with history of 2 months' amenorrhea with urine pregnancy test being positive. She has been reporting decreased sleep and feels anxious about being on medication and its effects on the infant. She wants to know whether she can stop medication. What would be the appropriate advice?**

The effects of antidepressants on the fetus can be broadly classified as teratogenic risks, perinatal toxicity and effects on the neurobehavioral development of neonates. Most of the retrospective studies which have studied SSRIs in general have shown no difference in the occurrence of major malformations when compared to general population (2%-4%) except for paroxetine.[33] Among the four prospective studies, only one study has found a possible association between cardiovascular anomalies and the first-trimester exposure to fluoxetine.[34] Hence, when it comes to major malformations, the occurrence is similar to that in the general population.

Fluoxetine has been associated with perinatal adverse effects. Cardiac arrhythmias and low birth weight have been reported. Two studies which have looked at perinatal outcomes have reported increased risk of premature delivery and poor neonatal adaptation with neonatal withdrawal symptoms.[35] Most of the adverse effects were self limiting. When it comes to long-term neurodevelopmental effects, the results are inconclusive. However it is clear that the research suggesting a lack of adverse events on infants' neurocognitive development is much larger and methodologically better conducted than the studies showing possible unwanted cognitive and behavioral effects.

Considering the available information about the safety of the drug the risks and benefits of antidepressant use, an informed decision needs to be taken.

**A 26-year-old woman is diagnosed to be suffering from moderate depression with somatic syndrome for the past 2 months. She is 8 months pregnant and is on sertraline 150 mg per day. The gynecologist asks for an opinion during the 35th week of pregnancy about the possible side effects to be monitored in the infant.**

SSRIs have been reported to be associated with adverse perinatal outcomes. Neonates primarily display central nervous system, motor, respiratory, and gastrointestinal signs that are usually mild and disappear by 2 weeks of age. Poor neonatal adaptation including low birth weight and mild respiratory distress has been reported in up to 30% of women on any SSRI. The infants are also at increased risk of developing serotonergic symptoms (myoclonus, restlessness, jitteriness, tremor shivering, hyperreflexia and rigidity) resulting from serotonin overstimulation.[35] The severe syndrome that consists of seizures, dehydration, excessive weight loss and hyperpyrexia, which may require intubation, is rare in term infants. With sertraline, there have been a few case reports of transient tachypnea, reduced behavioral pain response and selective abnormalities in blood counts. The obstetrician should be informed to monitor for the above symptoms of neonatal adaptation syndrome.

**A 30–year-old woman presents with a history of recurrent depressive disorder and has remitted with paroxetine. She wants to conceive and wants to know whether paroxetine is safe during pregnancy.**

Recent studies have shown a possible association of major malformations with the use of paroxetine during pregnancy. Increased risk of both major malformations (omphalocele and craniosynostosis) and cardiac abnormalities (ventricular or atrial septum defects) has been documented in four retrospective studies.[33] Two prospective studies have shown a higher incidence of cardiovascular anomalies when compared to control groups.[36,34] But a recent meta-analysis has reported a nonsignificant difference in the occurrence of cardiac abnormalities when compared to the general population.[37] Even though these reports need to be confirmed by large prospective controlled investigations, they call for caution in prescribing such a drug in early pregnancy. In view of a possible association between paroxetine and cardiovascular anomalies, in the above clinical situation, the best decision would be changing over to a safer antidepressant. Options include another SSRI, a tricyclic, or one of the newer agents which are discussed below.

**A 30–year-old woman presents with history of severe depression with inadequate response to SSRI. Therefore, she was started on imipramine 225mg/day. She has been maintaining well with the above medication for a period of one year and has come with a history of 3 months' amenorrhea. Her urine pregnancy test has come positive. Patient wants to know whether she can continue pregnancy and if so whether she can stop medication as she has been well for a year.**

Prenatal use of tricyclic antidepressants in humans has not been associated with congenital anomalies, although there is sparse literature in this area.[38] There have been reports that these medications may produce transient neonatal toxicity or withdrawal symptoms when used near the time of delivery. These may be in the form of lethargy, hypotonia, and anticholinergic effects such as constipation, tachycardia, and urinary retention. Hence, nortriptyline and desipramine are preferred over other tricyclic antidepressants during pregnancy as they have lower likelihood of anticholinergic and hypotensive side effects. Though studies on tricyclics are less, with the available information the perinatal adverse affects are transient. Since the exposure has happened, it is advisable to get an anomaly scan at 16–19 weeks

When it comes to discontinuing or changing of antidepressants in the above case, the risks of relapse needs to be discussed and informed decision to be taken.

**A 22-year-old woman presented with history of feeling sad and not being able to sleep at night for the past one month. Patient has delivered a female child 2 months back. She also feels less emotional with the child, frequently cries and feels guilty about herself for giving birth to a female child. Patient was prescribed escitalopram 10 mg. Family were concerned whether patient can breastfeed her infant.**

At present with the available literature, sertraline and paroxetine should be the first line drugs for depression in women who are breast feeding. Escitalopram is the biologically active S-enantiomer of citalopram. With citalopram, there have been reports of colic, irritability and delay in development in two infants exposed to the drug, but these were transient.[39] Recently, it was confirmed that escitalopram is excreted into breast milk and the total relative infant dose for escitalopram plus its metabolite was calculated to be 5.3%, which was lower than that of citalopram.[40] There are a couple of case reports of women using escitalopram during breast feeding which have not reported any adverse effects to the infants. Hence, escitalopram should be preferred over citalopram for the treatment of depression in breastfeeding women. In the above clinical situation it is advisable to continue and the infant needs to be carefully watched for any adverse effects.

**A woman aged 25 years has been on treatment for recurrent depression for the past year. During her last episode, she was treated with venlafaxine, 150 mg/day. Her urine pregnancy test is positive, and an ultrasonogram confirms the presence of a viable gestation of 12–14 weeks of age. She is concerned about the effects of the drug on the child, and wants to discontinue medications. What should she be told?**

A prospective study of 150 pregnant women conducted by the Motherisk Group in Canada[41] showed that venlafaxine was not associated with an elevated rate of congenital malformations. A meta-analysis of newer antidepressants – venlafaxine, reboxetine, mirtazapine, trazodone and nefazodone – found none to be associated with a higher rate of congenital malformations.[42] These results suggest that venlafaxine appears free of teratogenic effects. However, these results must be viewed with caution considering that venlafaxine at lower doses is serotonin-selective[43] and that animal models implicate serotonergic modulation in cardiac malformations.[44]

Venlafaxine and its metabolite have concentrations in neonatal cord blood similar to those in maternal serum.[45] A recent study[46] has described the occurrence of a behavioral syndrome in infants born to mothers receiving either venlafaxine or SSRIs; however, only one infant was exposed to venlafaxine. The authors found these symptoms to be transient, and more frequent to occur in premature infants. A larger retrospective study that examined newer antidepressants – including venlafaxine[47] – documented an elevated rate of neonatal complications, including preterm births, respiratory problems, low Apgar scores, hypoglycemia and seizures; these effects were as common as they were with SSRIs. A case report[48] also describes the occurrence of seizures in two neonates exposed to venlafaxine during the third trimester. Information on the safety of venlafaxine in breast-feeding is sparse. In this woman, the risks of relapse – with harmful consequences for both mother and baby – should be weighed against our uncertainty regarding the actual risk/benefit ration of venlafaxine. High-resolution ultrasonography at 18–20 weeks will help to detect congenital malformations as exposure has already happened

**A woman aged 30 years, recently married, has been suffering from generalized anxiety disorder for the past seven years. She was unable to tolerate two trials of SSRIs and is currently on a combination of buspirone 40 mg/day, chlordiazepoxide 12.5 mg/day and clonazepam 1 mg/day with which her symptoms are controlled. Currently, she wishes to conceive, but is worried that her symptoms may relapse if she stops medications. How would you proceed in this case?**

Generalized anxiety disorder (GAD) is twice as common in women as in men,[49] and a recent systematic review reported elevated rates in the perinatal period.[50] Given the chronic nature of her symptoms, any discussion regarding planning a pregnancy should include the risks of relapse of GAD and its potential effects on both her and her baby[51] and the treatment options available.

A meta-analysis[52] has shown that there is an association between benzodiazepine (BDZ) use and the development of malformations, in particular oral clefts. However, this was found only in case-control but not in cohort studies, raising the possibility of a recall bias. Other malformations reported include neural tube defects, limb defects, duodenal and anal atresia.[53] A review of the topic[54] concluded that while benzodiazepines do not appear to be major teratogens, high resolution ultrasonography should be done in case of exposure. Studies of individual drugs such as clonazepam[55] and chlordiazepoxide[56] were negative for teratogenicity. Combined use of SSRIs and benzodiazepines has been associated with congenital heart disease.[57]

There is a paucity of data on the safety of buspirone during pregnancy. There is also no information on its safety during breast-feeding, though the manufacturer's information states that the drug should be avoided during nursing if possible based on animal models. In this woman, it would be prudent to advise non-pharmacological interventions for treatment of GAD. If at all, a pharmacological agent has to be used, choose a drug that has data on safety. If benzodiazepines have to be used, then a long acting BDZ such as clonazepam can be used for short-term symptom relief.

**A woman aged 28 years, G3 P2, is currently in her 20^th^ week of gestation, and presents to you with the complaint of poor sleep. She asks for medications to help her sleep soundly. What would you recommend?**

Disorders of sleep are common during pregnancy have been described since the time of Hippocrates, and include daytime sleepiness, nocturnal awakening, insomnia, obstructive sleep apnea, and restless legs syndrome.[58,59] Though these disturbances are frequently dismissed as “normal,” they can trigger mood and anxiety disorders and disrupt interpersonal relationships, including mother-infant bonding.[59]

The evaluation and management of this woman's complaints should proceed systematically. Screening for comorbid psychiatric and general medications that can interfere with sleep – such as anxiety or mood disorders, pain, or gastro-esophageal reflux – should be carried out. As described above, the safety of most benzodiazepines in pregnancy is in doubt, and – more alarmingly – lorazepam, which is often used in general practice, has recently been associated with anal atresia in the neonate.[60]

Zolpidem, a selective agonist of benzodiazepine receptors has found no evidence of teratogenicity in animal models. Animal studies also suggest that, unlike benzodiazepines, Zolpidem has no enduring effects on post-natal behavior.[61] It is probably the drug of choice for short-term management of insomnia in pregnancy, but experience and evidence are lacking. Another small study[62,63] has suggested that zopiclone, a hypnotic belonging to the same class as zolpidem, is not associated with congenital malformations; it can thus be considered as an alternative to zolpidem. However, in a register-based study,[64] it was found that zolpidem and zopiclone may be associated with preterm delivery, low birth weight, and neonatal behavioral problems.

Even less is known about the safety of these drugs during lactation, though a small study indicates that the related drug zaleplon[65] disappears rapidly from milk and that feeding more than 1 hour after medication intake may be safe for the infant. Before offering pharmacotherapy, a trial of behavioral interventions should be tried.

**A woman aged 27 years developed major depression during the 2nd trimester of her pregnancy. Due to concerns about the harmful effects of serotonin reuptake inhibitors on her child, her family doctor has started her on mirtazapine, 30 mg/day, with which she achieved remission. She has just delivered a healthy male child weighing 3.2 kg and wants to know if it is safe for her to feed her child or if she should stop or change her medication. What would you tell her?**

The pharmacokinetics of mirtazapine during lactation have recently been investigated by Kristensen *et al.* (2006).[66] Both mirtazapine and its metabolite were detected in breast milk; however, the dose received by the infant relative to an adult dose was only 1.5%, and a detectable serum mirtazapine was found in only one of the infants. Based on this, the authors concluded that the drug was probably safe during lactation. Two case reports measuring serum levels[67,68] came to similar conclusions.

With this information in mind, and given the risk of exacerbation of depression in the puerperium, the benefits to the mother in this case probably outweigh the risk of harm to the infant, which appears low.

**A woman aged 34 years, G2 P1, was diagnosed to have somatoform pain disorder 2 years ago, and was on regular treatment with amitriptyline, 100 mg/day, with which her symptoms improved. As she wished to conceive again, the medication was tapered and stopped. However, after conceiving, her symptoms re-emerged during the 26th week of her gestation and are causing impairment in her day-to-day activities. Her physician has advised her to take duloxetine, 60 mg/day, as it is a newer drug with lesser side-effects compared to amitriptyline; however, she is apprehensive about whether it is safe for the child and has sought your opinion regarding the same. What would you do?**

Somatoform presentations during pregnancy, in the form of multiple physical complaints, have been described in the literature.[69]

The patient described above has experienced a relapse of pain-related complaints in the context of pregnancy and discontinuation of antidepressants. Therefore, before initiating any form of treatment, it is important to assess whether the current physical complaints are related to pregnancy or its complications, or represent a relapse of the prior somatoform disorder. In addition, comorbid psychiatric conditions (such as anxiety and depression) and general medical conditions (such as anemia and pregnancy-induced hypertension), which can present in a similar fashion, should be carefully looked for. If the diagnosis of somatoform pain disorder is confirmed, the treatment of choice would be brief psychological interventions, such the reattribution techniques described by Goldberg[70] and adapted to a “developing country” setting by Patel and Sumathipala (2007).[71]

If pharmacotherapy is to be used, the risks and benefits associated with it should be carefully discussed with the patient. No systematic study of the effects of duloxetine in pregnancy has been carried out. A single case report[72] describes poor neonatal adaptation in an infant born to a mother who had received duloxetine during pregnancy. There is no evidence regarding potential adverse effects of duloxetine during nursing, and the manufacturer's information has recommended that breast-feeding mothers avoid the drug. Given the greater base of clinical experience and evidence associated with tricyclic antidepressants (see the section above), cautious reintroduction of amitriptyline may prove to be a safer option for her.

## CONCLUSIONS

Psychiatric illnesses are common during pregnancy and post-partum period. Treatment of these conditions is important as they can affect the health of the mother and the fetus/infant. Issues become even more complex with multiple drugs and if there are associated medical problems or the infant is premature. Important issues to be kept in mind include folate supplementation in all women in the reproductive age group; planning pregnancies to minimize fetal exposure, discussion with patient and family regarding options and documentation of all discussions and decisions. Active liaison with obstetricians, ultrasonologists and pediatricians need to be stressed, as also the need for local registries. Whenever possible, use nonpharmacological approaches in addition. If a psychotropic is necessary, the choice of medication should be guided primarily by its safety data during pregnancy and breast-feeding and by the psychiatric history of the patient.
